# Nonextraction Treatment of Maxillary Canine and Lateral Incisor Transposition: A Case Report

**DOI:** 10.7759/cureus.63575

**Published:** 2024-07-01

**Authors:** Komal J Agrawal, Khyati Gupta, Prachi Khandelwal, Anushka Jain

**Affiliations:** 1 Department of Orthodontics and Dentofacial Orthopedics, Sharad Pawar Dental College and Hospital, Datta Meghe Institute of Higher Education and Research, Wardha, IND; 2 Department of Oral Pathology, Sharad Pawar Dental College and Hospital, Datta Meghe Institute of Higher Education and Research, Wardha, IND

**Keywords:** esthetics, lateral incisors, caine, dental anomalies, medicalization, transposition

## Abstract

The transposition of teeth is an infrequent dental abnormality characterized by the exchange of position between two adjacent teeth. This report presents a unique case of transposition involving the maxillary right canine and lateral incisor in a 20-year-old female patient. Clinical assessment and radiographic evaluation revealed the unconventional positioning of the affected teeth. Treatment planning necessitated collaborative efforts between orthodontic specialists to achieve optimal esthetic and functional outcomes. The patient underwent a comprehensive treatment regimen, including orthodontic alignment by distal drifting of the canine and mesial shifting of the lateral incisor. Subsequent long-term follow-ups confirmed stable occlusion and satisfactory esthetics in a time period of 18 months. This case underscores the significance of precise diagnosis and a multidisciplinary approach in managing intricate dental anomalies such as tooth transposition.

## Introduction

Transposition can be described as the swapping of the positions of two teeth in the same quadrant. It is very important to understand what might cause this aberration since dental transposition should be managed in the most effective manner and at the earliest time possible with the aim of achieving optimal esthetic and functional occlusion. According to the classification by Peck and Peck in 1995, it could be classified according to the teeth involved and is of six types. The common canine and lateral incisor transposition is seen in 20% of the total population in India [1]. Transposition should be treated because it enhances the look of the smile and the patient's contentment. Consequently, transposition, which is a type of dental malposition that involves the exchange of two teeth in terms of their position in the dental arches, poses a diagnostic and therapeutic challenge in orthodontics [2]. This means that the ideal position allows the reciprocal occlusion to be attained to optimize the masticatory function and minimize the prospects of developing temporomandibular disorders [3]. Crooked teeth are also hard to reach when it comes to brushing. This is especially true for the areas of the mouth where the gap is located, which results in an increased incidence of periodontal diseases and caries. It is also factual that malocclusion affects the speech, so correction of transposition can enhance the speech. When transpositions are not managed, they may result in wear facades, occlusion, and temporomandibular joint disorders. Transposition is a rare dental abnormality and causes few diagnostic and treatment issues in orthodontic practice.

This study will attempt to describe the case of a female patient who presented a canine and a lateral incisor transposition. A few proposed etiologies of tooth transposition include the following: it may be that the position of the tooth buds may change when they are still in development, the child may have suffered juvenile dental injuries, the sequence that teeth grow and develop may have interfered, or the presence of the deciduous teeth at the time when the permanent teeth are supposed to be growing. Because correction is time-consuming, the condition has to be diagnosed professionally at that stage and treated adequately. Consequently, the management of transposition can be definitive if only the type of transposition has been detected at an early age; otherwise, it can be interceptive if only at a later age. In orthopantomography and when assessing the position of the dental arches at the first stage of treatment, if there is a transposition of a tooth, children aged six to eight years are placed on intercepting treatment. In this therapy, all the remaining deciduous teeth are removed, and the permanent lateral incisor is placed in the right position; the site for the permanent canine is kept reserved. The next course of treatment should be the definitive treatment, which is the alignment of the teeth with the help of orthodontic options that will place the teeth in the right position. The control of anteroposterior and vertical dimensions of space is often difficult to evaluate and treat due to its effect on occlusion and facial aesthetics [4-6].

## Case presentation

**Figure 1:**
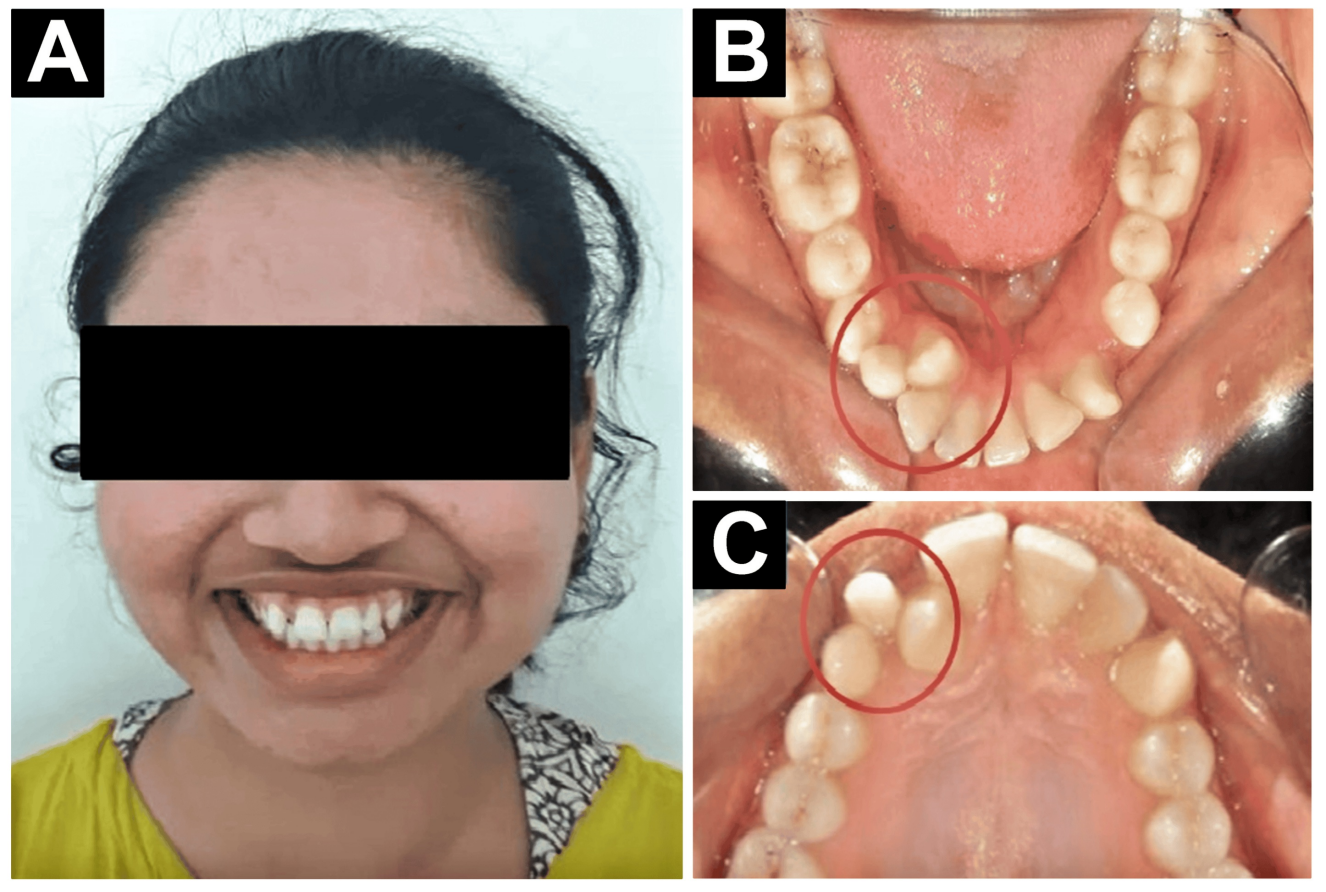
Pretreatment records. (A) Extraoral photograph. (B) Intraoral upper arch photograph. (C) Intraoral lower arch photograph. The marked areas show transposition between the canine and the lateral incisors

A female patient aged 20 years and 2 months visited the Department of Orthodontics with a chief complaint of misaligned upper front teeth and overretained deciduous canine. Upon evaluation, she was found to have a skeletal class I malocclusion with a hypodivergent growth pattern and Angle’s class I molar malocclusion. Additionally, she had a lateral incisor and canine transposition, as well as a peg-shaped lateral incisor. She also had normal overjet and overbite, overretained deciduous canine in the upper and lower right side, acute nasolabial angle, convex profile, and consonant smile arc. The patient was healthy, and on examination, the medical and dental history did not indicate any contraindication to dental treatment. On general examination, it was seen that there were potentially incompetent lips, protruding upper and lower incisors, and a protruding lower lip (Figure 1). It was revealed in the clinical examination that the patient had mixed dentition, along with a europrosopic facial form, a brachycephalic head profile, and a straight nose contour. In the upper arch, mesiobuccally rotated left canine with peg lateral on the right side, and transposition of canine and lateral are seen.

**Figure 2:**
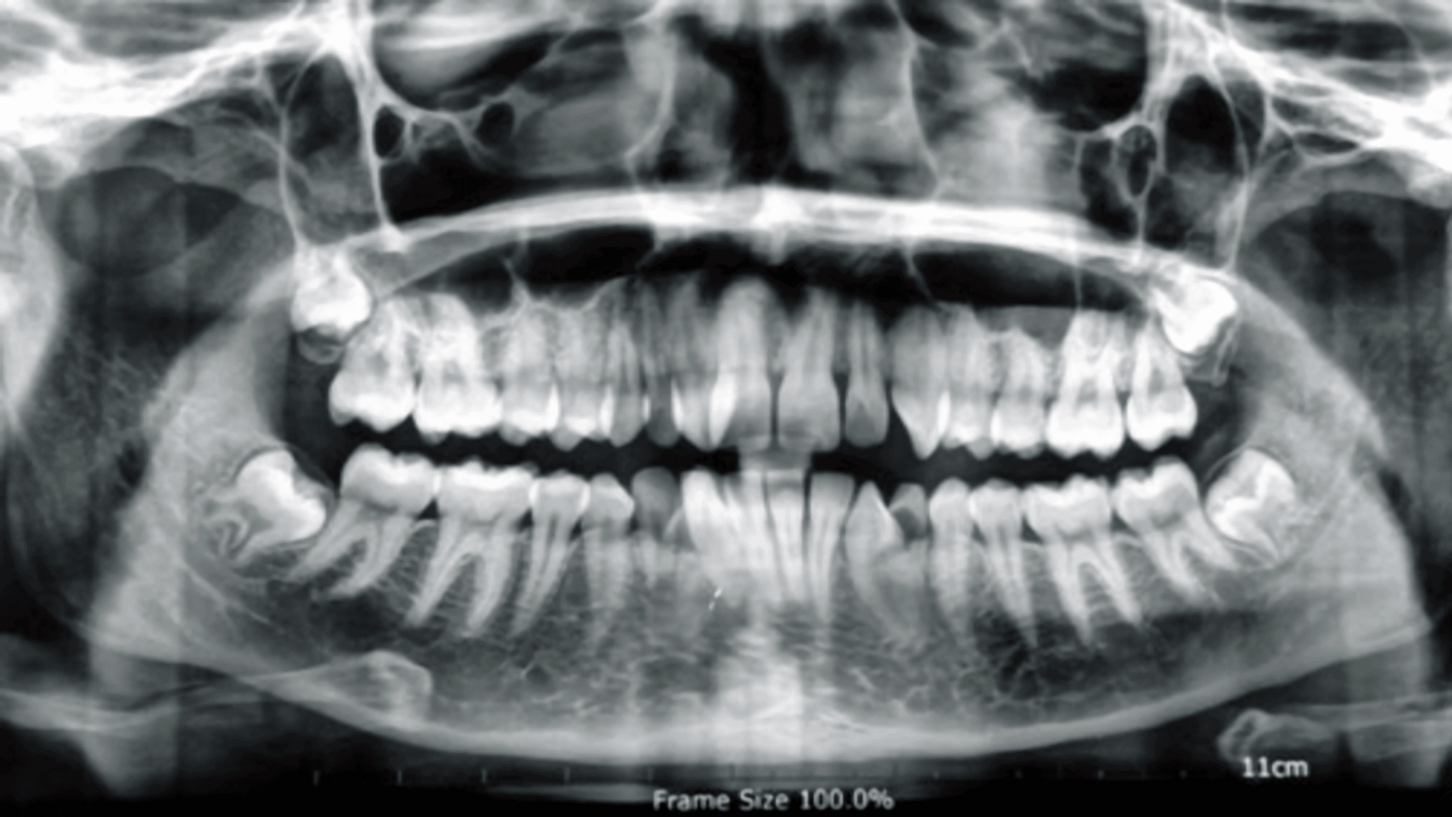
Pretreatment orthopantomogram

In the lower arch, a mild crowding of 2.5 mm, a 2 mm curve of the spherical bone, mesiobuccal rotation of the right lateral incisor, and distobuccal rotation of the left canine were found. All these findings can be appreciated in the orthopantomogram (Figure 2).

Cephalometric analysis 

**Figure 3:**
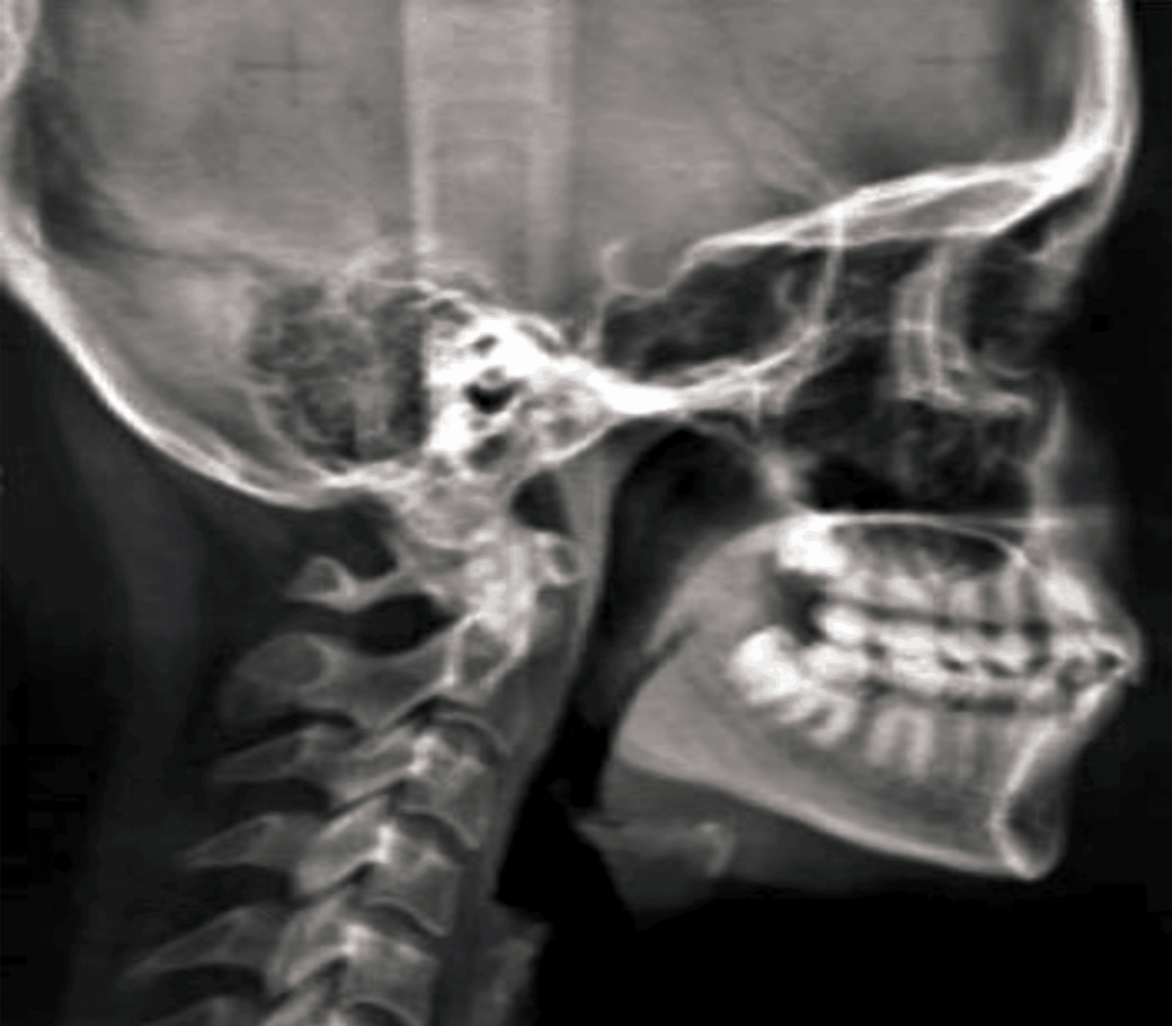
Pretreatment cephalometric analysis

In the pretreatment analysis of the patient, it was found that there is transposition between the canine and lateral incisor, with a protrusion in the upper anterior having skeletal class I malocclusion. The cephalometric analysis was done through the lateral cephalogram (Figure 3), and the pretreatment cephalometric findings (Tables 1, 2) were obtained.

**Table 1:** Skeletal parameters SNA: angle between the sella/nasion plane and the nasion/A plane; SNB: angle between the sella/nasion plane and the nasion/B plane; ANB: angle between point A of the maxilla and point B of the mandible; GO-GN to SN: angle between the gonion/gnathion plane and the sella/nasion plane

Skeletal parameter	Normal	Value
SNA	82°	82°
SNB	80°	81°
ANB	2°	1°
Nasion perpendicular to point A	0±2°	1°
Nasion perpendicular to pogonion	0-1°	-2°
Beta angle	27-35°	29°
Effective maxillary length	74.8 mm	71 mm
Effective mandibular length	18.67 mm	23 mm
GO-GN to SN	32°	24°
Frankfort-mandibular plane angle	25°	20°
Jarabak ratio	62-65	72
Bjork sum	396	385
Saddle angle	123±5°	119°
Gonion angle	128°	120°

**Table 2:** Dental parameters U1-NA: upper incisor to nasion-A measures; L1-NB: lower incisor to nasion-B measures; U1-L1: angle between the long axis of upper incisors to the long axis of lower incisors; U1-SN: angle between the long axis of upper incisors to the sella/nasion plane; L1-NB angle: angle between the long axis of lower incisors and nasion-B; L1-A pog: lower incisor to pogonion-A measures

Dental parameter	Normal	Value
U1-NA	4/22	8/32
L1-NB	4/25	6/31
Incisor mandibular plane angle	90°	104°
U1-L1	120°	116°
U1-SN	102°	115°
L1-NB	25°	31°
L1-A pog	1-2 mm	6 mm
Steiner line to the upper lip	-2 mm	1 mm
Steiner line to the lower lip	0 mm	5 mm
Nasolabial angle	900-100°	78°

Cone beam computed tomography

**Figure 4:**
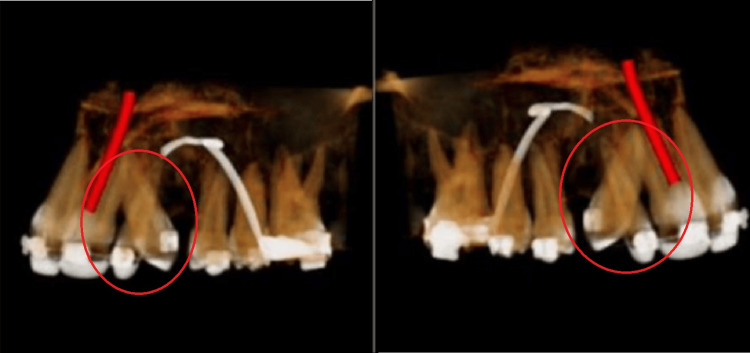
Pretreatment cone beam computed tomography. The marked area shows the transpositioned canine and lateral

In the patient's pretreatment cone beam computed tomography, it was appreciated that roots in the canine and lateral incisors had changed position. From this, it was concluded that there was a transposition in the canine and lateral incisors, as shown in Figure 4.

Treatment objectives

The treatment goals are to correct the jaw relationship, get molar and canine class I relations, correct the gummy smile, align the teeth, coincide the lower arch midline with the upper arch and facial midline, and improve lip competency, smile, and soft-tissue profile. 

Treatment plan

**Figure 5:**
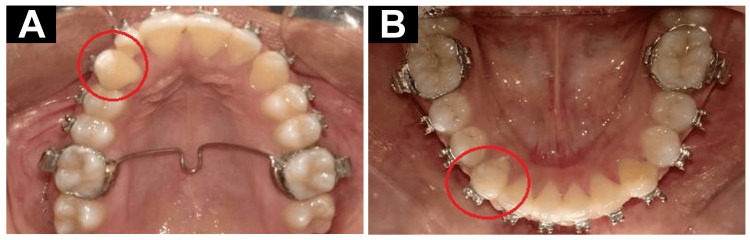
Posttreatment records. The marked areas show the corrections done. (A) Maxillary arch. (B) Mandibular arch

Once diagnosis had been completed, orthodontic treatment was planned. The treatment plan encompasses a targeted approach to address the transposition of teeth #12 and #13 and achieving proper canine occlusal alignment in the lower arch. This comprehensive strategy was presented to the patient and her guardians, who accepted it. After prophylaxis, the treatment procedure started with the extraction of retained deciduous teeth #53 and #83. In the upper arch, alignment with distalization of the upper canine was done with the help of ligature lace backs. After distalization, a canine 0.022 x 0.028 McLaughlin, Bennett, Trevisi bracket was placed. Later, a piggyback was placed for controlled buccal movement of the canine. Wire no. 16*22 stainless steel wire for the main arch and a 0.1014 NiTi segment arch-wire were used for attachment in the canine bracket. In the lower arch, a modified lingual arch was given for rotation correction of both canines. For cross-bite correction, the posterior bite plane was given. Supracrestal fiberotomies of 33 and 43 were done. The overall prognosis was good throughout the treatment. The final posttreatment outcomes can be seen in Figure 5.

## Discussion

Transposed lateral incisor-canine is a unique challenge that can arise in an orthodontist’s practice when planning treatment. In another study, it was found that dental transposition is more common in females than in males, and on the other hand, other authors have also noted a higher incidence of the said condition in males [7]. The present report reflects one of such outcomes in the framework of this rather atypical but clinically relevant pathology. The objective of this work is to expand the existing knowledge regarding this subject through a summary of the literature review, the description of the diagnostic process, the proposed treatment plan, and the outcomes regarding an orthodontic canine and lateral incisor transplantation in a female patient. Therefore, the present case report aims at raising the veil of further awareness and understanding of this complex dental disease to improve the management of other patients with such clinical features. But, while assessing the treatment plan in different clinical scenarios, it would be possible to state that there are different approaches for treating impacted canines and transpositions between the canine and the lateral incisor. Transpositions are often likely to need orthodontic treatment to facilitate the correction process. Surgical correction is needed in the case of severe transpositions or when orthodontic treatment is not enough. The basic management plan for the affected dogs requires the surgical mobilization of the canine and the orthodontic adjustment of the tooth in a position that is proper for occlusion [3]. In a case where surgical exposure cannot be done or when a canine is impacted and positioned unfavorably, extraction can be done with subsequent prosthetic restoration using either an implant or a fixed dental prosthesis [3]. Surgical repositioning, 4 out of 5, may help place the transposed teeth in their correct position and then fine-tune them with orthodontic treatment [8]. In cases where the canine is partly or significantly displaced, it is arguably correct to transplant the canine back to the right position [9]. They can provide additional support and assist the canine in walking situations where the canine is transposed or impacted. Understanding the various treatment plans and consulting with specialists will go a long way in enhancing the quality of treatment for patients diagnosed with such dental anomalies.

## Conclusions

The case report delineates an uncommon instance of tooth transportation occurring between the canine and the lateral incisor. The transportation event was correctly diagnosed by using extensive clinical assessments and diagnostic imaging. Treatment approaches were observed to prioritize achieving the best probable results for patients by attending to specific anatomical factors. This case emphasizes how important it is to manage dental anomalies with careful clinical analysis as well as customized treatment planning. By recording and sharing these cases, the dental community can improve its understanding and strategy for dealing with comparable clinical issues. 
